# Four European *Salmonella* Typhimurium datasets collected to develop WGS-based source attribution methods

**DOI:** 10.1038/s41597-020-0417-7

**Published:** 2020-03-03

**Authors:** Nanna Munck, Pimlapas Leekitcharoenphon, Eva Litrup, Rolf Kaas, Anika Meinen, Laurent Guillier, Yue Tang, Burkhard Malorny, Federica Palma, Maria Borowiak, Michèle Gourmelon, Sandra Simon, Sangeeta Banerji, Liljana Petrovska, Timothy J. Dallman, Tine Hald

**Affiliations:** 1 Research Group for Genomic Epidemiology, National Food Institute, Technical University of Denmark, Kgs. Lyngby, Denmark; 2 Foodborne Infections, Department of Bacteria, Parasites and Fungi, Statens Serum Institute, Copenhagen, Denmark; 3 Department for Infectious Disease Epidemiology, Robert Koch Institute, Berlin, Germany; 4 Université Paris Est, ANSES, Laboratory for Food Safety, F-94701 Maisons-Alfort, France; 5 Department of Bacteriology, Animal and Plant Health Agency, Weybridge, Surrey, UK; 6 Department of Biological Safety, German Federal Institute for Risk Assessment, Berlin, Germany; 7 Ifremer, Environment and Microbiology Laboratory, RBE, SGMM, Plouzané, France; 8 National Reference Center for Salmonella and other bacterial enteric pathogens, Robert Koch Institute, Wernigerode, Germany; 9 National Infections Service, Public Health England, London, UK

**Keywords:** DNA sequencing, Bacteriology, Bacterial infection

## Abstract

Zoonotic *Salmonella* causes millions of human salmonellosis infections worldwide each year. Information about the source of the bacteria guides risk managers on control and preventive strategies. Source attribution is the effort to quantify the number of sporadic human cases of a specific illness to specific sources and animal reservoirs. Source attribution methods for *Salmonella* have so far been based on traditional wet-lab typing methods. With the change to whole genome sequencing there is a need to develop new methods for source attribution based on sequencing data. Four European datasets collected in Denmark (DK), Germany (DE), the United Kingdom (UK) and France (FR) are presented in this descriptor. The datasets contain sequenced samples of *Salmonella* Typhimurium and its monophasic variants isolated from human, food, animal and the environment. The objective of the datasets was either to attribute the human salmonellosis cases to animal reservoirs or to investigate contamination of the environment by attributing the environmental isolates to different animal reservoirs.

## Abstract (summary)

**Table:** 

Measurement(s)	sequence_assembly • DNA
Technology Type(s)	sequence assembly process • DNA sequencing • Paired-End Sequencing
Factor Type(s)	geographic location • Salmonella Typhimurium isolate source
Sample Characteristic - Organism	Salmonella
Sample Characteristic - Location	Kingdom of Denmark • Germany • United Kingdom • French Republic

Machine-accessible metadata file describing the reported data: 10.6084/m9.figshare.11816748

## Background & Summary

The datasets described in this descriptor were collected in the context of Work Package 4/7 of the Collaborative Management Platform for detection and Analyses of (Re-) emerging and foodborne outbreaks in Europe (COMPARE, Horizon2020 research project grant number 643476). This research network aims to develop general analytical workflows for population-based disease surveillance, outbreak detection and epidemiological modeling of foodborne infections. A specific task therein was to develop methods for source attribution applying whole genome sequencing (WGS)-based surveillance and outbreak data for foodborne pathogens. In brief, source attribution models estimate the number and/or proportion of (human) cases of a specific foodborne illness that can be attributed to specific food categories and animal reservoirs^1^. Source attribution models require data from humans and potential sources that are: (1) representative of what the human population is exposed to, (2) related in time and space^2^, (3) harmonized regarding categorization of the sources^1^ and (4) analysed using a discriminatory subtyping method^1^. Data collected through integrated surveillance systems of humans, food and animals complies with these requirements^2^.

The potential of applying sequence data for source attribution purposes based on a machine learning approach has recently been reported^3,4^ and discussed^5^. These agree on the potential of the machine learning method to discriminate between different sources and applicability to trace foodborne outbreaks. In comparison to the datasets used by Zhang *et al*. (2019) and Lupolova *et al*. (2018), our datasets consist of food and animal data collected in a narrow time frame (3–5 years) and specific geographical area (country) and to the extent possible include a large representative sample of all human *Salmonella* Typhimurium infections reported in the country in the same time period. With such datasets, we believe we can make inferences on the relative contribution of the included sources (food and animals) to the number of human infections in the study period. In the study by Zhang *et al*. (2019), 51 human cases were attributed (or predicted) to animal reservoirs. However, the isolates from food, animals and humans were collected over a much wider time span and therefore more appropriate for studying the evolution of *Salmonella* Typhimurium in livestock sources and the relation to humans, than for quantifying the contribution of specific sources to human infections occurring in a shorter time period. If applied regularly on surveillance data, such quantification of the contribution of specific sources to human infections can be used to inform food-safety decision-making and to monitor the effect of control initiatives.

**Fig. 1:**
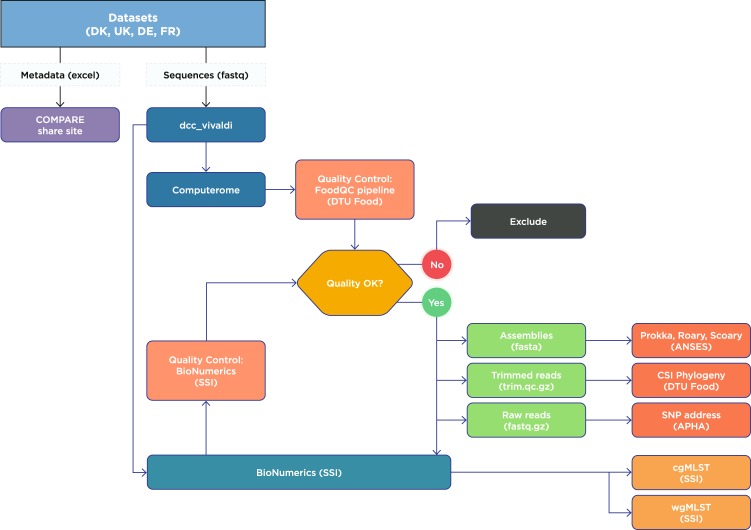
Flow chart of data generation, data management and quality control. dcc_vivaldi^8^: a private datahub (https://www.ebi.ac.uk/ena/pathogens/login) set up for data sharing and hosted at the European Nucleotide Archive (ENA). Computerome: a local server used by DTU Food (https://www.computerome.dk). Prokka: Rapid Prokaryotic genome annotation^32^. Roary: Rapid prokaryotic genome annotation^33^. cgMLST and wgMLST obtained using the Enterobase scheme^11^ in BioNumerics version 7.6 (Applied Maths, Sint Martens Latem, Belgium).

A benchmarking study was established to venture further into this subject and explore and assess different bioinformatics analyses and source attribution models based on sequencing data and to provide recommendations on how to evaluate and select the best approach for a given dataset. The results of the benchmark study will be reported elsewhere. In this paper, we describe in detail the four datasets that were applied including the sampling frameworks, metadata, sequencing techniques and quality control analyses (Fig. 1). Three of the datasets, representing data from Denmark, Germany and the United Kingdom, consist of strains of *Salmonella* enterica serovar Typhimurium incl. monophasic variants from humans and different animal reservoirs and food including pigs/pork, cattle/beef, broilers/chicken meat and laying hens. The fourth dataset from France includes a selection of environmental strains as well as strains from animal reservoirs. A total of 1,781 strains from humans (n = 943), animals (n = 804) and environment (n = 34) were collected as part of this study (Table 1). A number of the UK human data was outbreak cases and therefore excluded.

**Table 1:** Overview of data collected and sequence quality. SA: Source attribution. BN: BioNumerics.

Dataset	Years included	Total sequences collected for the study	Poor quality			Excluded for other reasons	Included in SA study
		n total = 1781 n humans = 943 n animal = 804 n environment = 34	FoodQC	BN QC	FoodQC + BN QC		n total = 1259 (n animal = 753, n human = 479, n environment = 29)
DK food	2013, 2014	211	1	0	0	0	210
DK human	2013, 2014	181	0	40	0	0	141
UK Food	2014, 2015, 2016	329	4	41	0	2	282
UK human	2014, 2015, 2016	596	29	273	0	117	177
DE food	2014, 2015, 2016	193	0	3	0	0	190
DE human	2014, 2015, 2016	166	0	5	0	0	161
FR animal	2010–2015	71	0	2	0	0	69
FR environmental	2010–2015	34	0	0	5	0	29

As the use of genomics data for source attribution is still nascent^6^ we consider the datasets valuable for other researchers who seek to explore new approaches or for comparing the performance of their own models and datasets with those presented here. Partners involved in this project were microbiologists, epidemiologists and bioinformaticians from the following institutes: The National Food Institute (DTU Food), Statens Serum Institute (SSI), Robert Koch Institute (RKI), German Federal Institute for Risk Assessment (BfR), French Agency for Food, Environmental and Occupational Health & Safety (ANSES), French Research Institute for Exploitation of the Sea (Ifremer), University of Bologna (UNIBO), Animal & Plant Health Agency (APHA), and Public Health England (PHE).

## Methods

### Sampling procedure

A representative set of sequenced isolates of *Salmonella* Typhimurium strains, including its monophasic variants, from humans and different environmental and animal sources, locations and years were collected from four different countries: Denmark, France, Germany, and the United Kingdom. The isolates were available through national surveillance/monitoring/control programs/research projects or larger surveys conducted between 2010 and 2016. Isolates from animals and food represent the major food animal reservoirs and thus reflect what humans are exposed to through consumption of food. Only one isolate per farm or food batch was included, and clinical isolates of animals were not considered. As a minimum, the following animal species and food types were covered: broilers/chicken meat (fresh), pigs/pork (fresh) and cattle/beef (fresh). The minimum sample size was initially set to 25 isolates per animal species per country per year^7^. However, this number of isolates was not always available, due to low *Salmonella* occurrence and low sample size of some sources. If available, isolates from other animal species and/or their related meat type, from fruit and vegetables and environment were included as well with a minimum sample size of 10 samples per category per country per year. The minimum sample size for humans was 100 isolates per country per year.

### Data sharing

#### Metadata

Metadata was shared among partner institutions via a database in Microsoft Excel format set up for the purpose and shared via the COMPARE share site. Dropdown menus defining categories for variables that could be standardized, such as “Primary source” and “Patient travel”, were set up using the Microsoft Excel function “Data validation” facilitating standardized registration of metadata. Each partner added sequence information and epidemiological data to the database (Table 2). A unique “sample ID” was provided for each sample. Validation of metadata was performed by DTU by assuring data was added in the correct format. In case of errors, the data provider was asked for correction.

**Table 2:** Metadata variables.

Sequence information
sample ID
Data provider
Pathogen Organism
Taxonomic Name/Serovar
Year of sampling
Primary Source
Imported Food (if relevant)
Country of sample origin
Outbreak ID (if relevant)
Patient Travel
MLST

#### Sequences

Raw reads were shared in a private datahub called dcc_Vivaldi^8^ (https://www.ebi.ac.uk/ena/pathogens/login) set up for the purpose and hosted at the European Nucleotide Archive (ENA). An accession number was linked to the single sequences upon uploading to ENA. Personal access credentials to dcc_Vivaldi datahub were granted by ENA whenever consent was obtained from a representative of each institution involved in the COMPARE Work Package 4/7. A subset of sequences was already publicly available and obtained from the Sequence Read Archive (SRA), these are designated with SRR IDs. Metadata and sequences were linked via the unique sample ID and accession number.

Before analyzing, the sequence data was transferred from the dcc_Vivaldi datahub to a local server such as Computerome (https://www.computerome.dk) used by DTU Food. Transferring these data required a stable internet connection during the transfer. All isolates were sequenced using Illumina chemistry producing paired end reads.

#### Assemblies

Assemblies were generated by the in-house software called FoodQCPipeline (https://bitbucket.org/RolfKaas/foodqcpipeline) and shared on a password-protected FTP server set up by DTU Food. Reads were *de novo* assembled using SPAdes 3.11.0^9^ in last step of the pipeline. FoodQCPipeline trimmed the raw reads using bbduk2 (part of BBMap version 36.49, https://jgi.doe.gov/data-and-tools/bbtools/) according to the following: (1) length of read must be higher or equal to 50 base pairs (bp), otherwise were excluded, (2) phred score per base higher or equal to 20 and (3) filter away adapters based on an internal database with Illumina adapters that was created and maintained by DTU Food. FastQC^10^ version 0.11.5 was applied to the reads before and after trimming generating a quality control report for every sample. The quality of the de novo assemblies were assessed using Quast version 4.5.

Core genome Multi-locus sequence typing (cgMLST) and whole genome Multi-locus sequence typing (wgMLST) analysis were used to generate input to different source attribution methods developed in the COMPARE project. cgMLST and wgMLST were obtained using the Enterobase scheme^11^ in BioNumerics version 7.6 (Applied Maths, Sint Martens Latem, Belgium). The cgMLST is based on 3,002 loci and the wgMLST on 21,065 loci with one single locus having several allele variations^11^. cgMLST allele calls were accepted for strains with a core genome coverage higher than 95% (2,852) of 3,002 core genomes alleles and a detection of mixed sequence alleles lower than 50 alleles.

#### Quality control

A 2-step quality control was applied to all sequences. First, outcomes from the FoodQC pipeline were assessed. Secondly, outcomes resulting from the quality control that is part of BioNumerics^11^ were assessed. The applied inclusion criteria are listed below.

From the FoodQC pipeline the following were assessed (1) fewer than 500 contigs (where contigs are >500 bp each), with an N50 value preferable larger than 30,000 bp, (2) total number of base pairs in contigs larger than 500 bp summed to approximately 5,000,000 according to the size of the *Salmonella* bacterial genome, (3) depth of coverage equal to or higher than 30x calculated as (base pairs sequenced)/(base pairs in assembly (from (2)) and (4) phred score equal to or higher than 20, which is calculated as part of the trimming process.

From BioNumerics the following were assessed based on the cgMLST calls (5) core genome coverage higher than 95% of 3,002 core genomes alleles and (6) mixed sequence alleles detected lower than 50 alleles.

#### Final datasets

All sequences with acceptable quality were included in the final datasets. Table 1 outlines the sequences collected, number of isolates that passed the quality control and the final datasets included in the study.

#### Applicability of the datasets for source attribution purposes

The phylogeny of all four individual datasets was analysed in order to examine the applicability of the four different datasets to develop new source attribution models. Datasets were assumed applicable when human salmonellosis cases or environmental strains were intermixed with the food and animal sources. Maximum likelihood phylogenetic trees were constructed from sequence variations in the genome shared between strains included in the given analysis using FastTree (gtr + cat model^12^). Sequence variations were defined as the single nucleotide polymorphisms (SNPs) within the genome shared between the strains included in the given analysis. SNPs were identified using the CSI phylogeny^13,14^, freely available from the Center for Genomic Epidemiology (www.genomicepidemiology.org) and described in more details in the following. Trimmed paired-end reads of each isolate included in the given analysis were aligned against the COMPARE reference genome, *Salmonella* enterica subsp. enterica serovar Typhimurium str. LT2 (AE006468.2, 4,857,432 base pairs)^15^ using Burrows-Wheeler Aligner (BWA) version 0.7.2^16^. The SNPs were identified using ‘mpileup’ module in SAMtools version 0.1.18^17^. SNPs fulfilling the following criteria were selected: (1) a minimum distance of 15 bps between each SNP (pruning), (2) a minimum of 10% of the average depth, (3) mapping quality above 30, (4) the SNP quality was more than 20, and (5) all indels were excluded. The selected SNPs from each genome were concatenated into a single pseudo alignment corresponding to the position of the reference genome. Phylogenetic trees were annotated and visualized using iTOL^18^ and distances between isolates equivalent to the amount of SNPs between them.

#### Communication

The datasets including the metadata, quality control criteria and strategies for sharing of data were continuously discussed at regular face-to-face meetings. In between the face-to-face meetings, continuous email dialogue and tele conferences were held to inform about progress and clarify any misunderstandings.

### Data and sampling

This section lists the reasons for choosing *Salmonella* Typhimurium and its monophasic variants as study organism and describes the sampling plan for all four European datasets. The Danish, German and British datasets were collected with the objective of attributing the number of reported sporadic human salmonellosis cases to animal reservoirs and food. The French dataset was collected with the objective of attributing environmental *Salmonella* Typhimurium strains to animal reservoirs.

#### Data

*Salmonella* Typhimurium including its monophasic variants is the second most prevalent serotype in humans in EU and most EU member states^19^. During the last few years, monophasic variants of *Salmonella* Typhimurium have been dominating human cases and have also repeatedly been involved in food-borne outbreaks^20^. *Salmonella* Typhimurium is a major serotype in pigs, but is also commonly found in a number of other food-animal reservoirs (e.g. poultry and cattle) and environmental samples. This is in contrast to other *Salmonella* serotypes such as *Salmonella* Enteritidis and *Salmonella* Dublin which are mainly associated with poultry and bovines, respectively. It was, therefore, decided to focus on attribution of human infections caused by *Salmonella* Typhimurium and its monophasic variants.

### Data sampling Denmark

#### Samples from human surveillance included in this dataset

Clinical cases of *Salmonella* in humans in Denmark are notifiable through the laboratory surveillance systems at Statens Serum Institut (SSI). SSI receives isolates from the Danish hospitals and is responsible for the pheno- and genotyping of clinical *Salmonella* isolates. Information regarding travelling abroad before disease onset was obtained from either travel interviews or general practitioner, and information about outbreak cases was available from SSI and registered in the Food- and Waterborne Outbreak Database^21^.

#### Samples from food and animal surveillance included in this dataset

All major food animals and food of animal origin are monitored for *Salmonella* through national surveillance programs. In addition, results from centrally coordinated studies supplement the surveillance programs, particularly regarding data on imported food of animal origin. The surveillance and monitoring programs are regularly revised and their contents are described in detail in the Annual Reports on Zoonoses in Denmark^22,23^. The following describes the content of the programs as they were during the years 2013–2014. Samples from animal and food were analysed at authorized private laboratories, the Danish Food and Veterinary Administration’s laboratory and the Technical University of Denmark (DTU). The National Food Institute at DTU performed serotyping, WGS and antimicrobial resistance testing.

Every commercial flock of layers was tested every 9 weeks before 1/10/2013 and every two weeks thereafter. All commercial flocks of broilers were tested two times at approximately three weeks and again one week before slaughter. All commercial flocks of turkeys and ducks were tested approximately three weeks before slaughter. There were no samples from flocks of ducks available in 2014. Every batch of broiler carcasses was tested after slaughter by the examination of a pool of neck skin samples. Pork and beef were sampled as pooled carcass swabs. The number of samples collected from each slaughterhouse was proportional to the number of animals slaughtered. Samples of imported pork and chicken meat were collected randomly at importers’ premises throughout the year. Finally, through centrally coordinated surveys, samples of both imported and Danish beef and duck meat were obtained at the retail level. All *Salmonella* surveillance and monitoring programmes, from which the reported data originates, are described in the Annual Report on Zoonoses in Denmark^22,23^.

In total, 325 samples of *Salmonella* Typhimurium and its monophasic variants isolated from domestic and imported food and animals were collected via the national *Salmonella* surveillance programs for animals and food during 2013 and 2014. Of these, 65% were available for sequencing and thus included in the dataset for this specific study. In total, 764 samples of *Salmonella* Typhimurium and its monophasic variants were isolated from humans in 2013 and 2014. Of these, 18% were available for sequencing and included in the human dataset for this specific study. All isolates were sequenced using an Illumina HiSeq, NextSeq or MiSeq sequencing machine. The isolates in the Danish dataset presented here originate from a quite intensive sampling as described above and the dataset per se is thus representative of *Salmonella* Typhimurium and its monophasic variants. Few isolates from Danish cattle and layer flocks reflects a very low prevalence.

This Danish dataset was used to attribute the number of reported human salmonellosis cases to animal reservoirs and food.

### Data sampling Germany

#### Samples from human surveillance included in this dataset

In Germany, the detection of *Salmonella* indicating an acute human salmonellosis is notifiable. Laboratories have to report this diagnostic finding to the respective local health authority of the district where the patient lives. Local health authorities then forward all the information relevant for surveillance via the state health authorities to the Robert Koch Institute as the national public health institute. Laboratories can also send a *Salmonella* isolate to the National Reference Center (NRC) for *Salmonella* and other bacterial enteric pathogens which is located at the Robert Koch Institute in Wernigerode, Germany, for further typing. However, forwarding of *Salmonella* isolates to the NRC is not mandatory. The NRC estimates receiving about 20% of isolates of all notified cases. Of all the *Salmonella* Typhimurium or the monophasic variants confirmed isolates which have been submitted to the NRC between the years 2014 and 2016, a random selection was chosen: 100 human isolates per year. For known outbreaks, only one isolate per outbreak was included.

#### Samples from food and animal monitoring included in this dataset

Food and animal isolates which originate either from official sampling or companies’ self-monitoring can be send to the National Reference Laboratory for the Analysis and Testing of Zoonoses (NRL *Salmonella*) located at the German Federal Institute for Risk Assessment (BfR) for further typing. Sending is not mandatory which means that the isolates at the NRL are not necessarily representative for all food and animal isolates in Germany. In accordance to the human isolates, a random selection was chosen according to the minimum sample size per source and year (25 isolates). Isolates originating from the same farm were excluded.

After checking the comparability of sequence quality between NRC and NRL, food and animal isolates were sequenced at the BfR. For library preparation, the Nextera XT kit was used and sequencing was performed on an Illumina MiSeq benchtop sequencer. Sequence data were then send to the NRC for further analysis.

This German dataset was used to attribute the number of reported human salmonellosis cases to animal reservoirs and food.

### Data sampling UK

#### Samples from human surveillance included in this dataset

Sequences from 177 *Salmonella* Typhimurium isolates from human infections collected and sequenced by Public Health England as part of the notifiable routine surveillance of human infections through the National Health Service were included in this study. Information regarding foreign travel before disease onset was obtained based on declaration from the sending physician.

#### Samples from food monitoring included in this dataset

The Animal and Plant Health Agency (APHA) receives reports of all *Salmonella* isolates from cattle, deer, goats, horses, pigs, rabbits, sheep, chickens, turkeys, ducks, geese, guinea fowl, partridges, pheasants, pigeons and quail, as required by the Zoonoses Order 1989 (http://www.legislation.gov.uk/uksi/1989/285/made). Under the 1989 Order the responsibility for reporting the isolation of *Salmonella* is placed on the laboratory carrying out the examination or in the case of examinations elsewhere, the person carrying out the examination. In practice, all reports of *Salmonella* isolations must be made to a Veterinary Officer at one of the Veterinary Investigation Centres (VICs) of the Animal and Plant Health Agency (APHA) or to a Regional Veterinary Lead in Scotland. A culture of the organism must be made available. Many isolations of *Salmonella* from livestock are not associated with clinical disease or occur on farm premises where *Salmonella* has been isolated from a group of animals rather than an individual. Further information on the reporting of *Salmonella*, *Salmonella* culture methods, phage typing and antimicrobial sensitivity testing methods is available in The *Salmonella* in Livestock production in GB reports available on the APHA website: https://www.gov.uk/government/publications/salmonella-in-livestock-production-in-great-britain-2017.

The objective of this dataset was to attribute the number of reported human salmonellosis cases to animal reservoirs and food.

### Data sampling France

#### Samples from food and animal monitoring included in this dataset

The dataset of strains used to characterize reservoirs mainly comes from a targeted national epidemiological surveillance system, called “The *Salmonella* network” and coordinated by ANSES’s Laboratory of Food Safety (based in Maisons-Alfort). “The *Salmonella* network” was established in 1997 in order to monitor *Salmonella* strains of non-human origin isolated from all stages of the food chain^24^. *Salmonella* strains and serotyping data are submitted to ANSES on a voluntary basis from public and private laboratories spread across the whole country. In the ANSES’s laboratories, serological and/or molecular typing (e.g. PCR, MLVA, PFGE and sequencing for the characterization of *Salmonella* Typhimurium variants) are performed on *Salmonella* strains, which are isolated from a variety of matrices such as sick and healthy animals, human food and feed and environments (natural environment, farm and processing plants). The typing data are generally associated with epidemiological data (e.g. country of isolation, the sample type, the sampling site, the context of isolation, etc.).

For the aim of this study, a dataset of 69 strains of *Salmonella* Typhimurium and its monophasic variants, isolated from different reservoirs between 2010–2014, were collected and whole genome sequenced.

A total of 49 strains of *Salmonella* were isolated from pigs, 14 from poultry (layers, broilers, turkeys and ducks) and 6 from ruminants (cattle, sheep and goat).

#### Samples from environment included in this dataset

Twenty eight (28) strains of *Salmonella* were isolated from environment (from fresh or brackish water as well as soil isolates) from both ANSES and IFREMER with the collaboration of University of Caen (France). The 15 IFREMER’s environmental isolates originate from a research project^25^. Institute Pasteur (France) determined the serotype (isolates from IFREMER). ANSES environmental isolates have been collected by the *Salmonella* network’s passive surveillance. They were isolated from soils (three strains) and fresh water (ten strains).

One additional strain isolated in crustacean (specifically, shellfish) was associated to the environmental dataset.

The objective of this French dataset was to attribute environmental *Salmonella* Typhimurium strains and its monophasic variants to animal reservoirs.

## Data Records

The final datasets are described in Tables 3, 4, 5 and 6. All sequences included are available for download through ENA (DK dataset^26–28^, DE dataset^27^, FR dataset^27^ and UK animal data^29^) and SRA (UK human^30^), where they are located. Sequences from SRA were publicly available before the beginning of this study. Assemblies, associated metadata and quality control of sequences with acceptable quality are available at figshare^31^.

**Table 3:** Number of *Salmonella* Typhimurium and its monophasic variants included in the Danish dataset.

DK dataset, 2013–2014 Source	2013	2014	Number of isolates
Pigs (DK)	84	41	125
Pigs (Import)	20	14	34
Broilers (DK)	13	21	34
Ducks (Import)	0	11	11
Layers (DK)	3	1	4
Cattle (DK)	1	0	1
Cattle (Import)	0	1	1
Total animal	121	89	210
Human	29	112	141

**Table 4:** Number of *Salmonella* Typhimurium and its monophasic variants included in the German dataset.

DE dataset, 2014–2016 Source	2014	2015	2016	Number of isolates
Birds (DE)	0	0	1	1
Broilers (DE)	5	4	1	10
Cattle (DE)	22	26	21	69
Game (DE)	1	0	0	1
Layers (DE)	5	20	12	37
Pigs (DE)	25	26	21	72
Total animal	58	76	56	190
Human	43	49	69	161

**Table 5:** Number of *Salmonella* Typhimurium and its monophasic variants included in the British dataset.

UK dataset, 2014–2016 Source	2014	2015	2016	Number of isolates
Broilers (UK)	4	2	3	9
Cattle (UK)	11	2	7	20
Game (UK)	3	9	6	18
Layers (UK)	4	2	1	7
Other mammals (UK)	14	8	20	42
Pigs (UK)	71	69	23	163
Reptiles (UK)	0	1	1	2
Sheep (UK)	0	1	6	7
Turkey (UK)	9	2	3	14
Total animal	116	96	70	282
Human	53	118	6	177

**Table 6:** Number of *Salmonella* Typhimurium and its monophasic variants included in the French dataset.

FR dataset, 2010–2015 Source	2010	2011	2012	2013	2014	2015	Number of isolates
Broilers (FR)	0	1	4	0	2	0	7
Cattle (FR)	1	0	1	2	1	0	5
Ducks (FR)	0	1	0	0	1	0	2
Layers (FR)	0	0	2	0	0	0	2
Pigs (FR)	0	16	21	6	6	0	49
Sheep goat (FR)	0	1	0	0	0	0	1
Turkey (FR)	0	0	0	0	3	0	3
Total animal	1	19	28	8	13	0	69
Environmental	1	2	1	11	13	1	29

## Technical Validation

All sequences of the four individual datasets were quality controlled to confirm the quality of the sequences further described in the Methods section. Isolates for which the quality was unacceptable (n = 403) were excluded from further studies as were isolates excluded for other reasons (n = 119). Output of the quality control per isolate with acceptable quality (n = 1,259) are included in the files at figshare^31^. Mean, minimum and maximum value of parameters of interest stratified for the different datasets are included in Table 7.

**Table 7:** Quality control criteria of interest for all datasets. Depth of coverage is calculated as bases(MB)*1,000,000/total base pairs. Mean (minimum value; maximum value) reported.

Data	N50	No. contigs	Total base pairs of assembly	Depth of coverage
DK human	274,445 (148,864; 490,821)	63 (35; 151)	4,932,862 (4,730,908; 5,217,740)	90 (46; 296)
DK food and animal	280,405 (151,473; 507,939)	60 (36; 184)	4,945,037 (4,769,818; 5,254,474)	104 (65; 154)
DE human	299,886 (188,812; 393,902)	61 (33; 326)	4,937,911 (4,682,559; 5,162,175)	103 (45; 276)
DE food and animal	137,544 (19,719*; 377,001)	112 (35; 466)	4,937,743 (4,730,293; 5,240,180)	79 (33; 173)
UK human	211,889 (53,936; 550,741)	69 (36; 170)	4,941,438 (4,723,064; 5,284,498)	74 (36; 178)
UK food and animal	267,335 (143,510; 464,171)	68 (31; 125)	5,037,113 (4,804,529; 8,982,655**)	83 (31; 215)
FR enviromental	263,923 (143,153; 356,020)	64 (42; 101)	4,939,325 (4,771,190; 5,090,477)	236 (56; 473)
FR animal	261,383 (143,153; 356,020)	64 (42; 89)	4,935,805 (4,810,678; 5,084,053)	215 (56; 431)

*This sequence was included despite its relatively low N50 value because the number of contigs and total base pairs were acceptable.

**This sequence was included despite its large size because the associated number of contigs and N50 value were acceptable and no contamination was observed from a kmer analysis using KmerFinder freely available from the Center for Genomic Epidemiology (www.genomicepidemiology.org).

### Phylogeny

**Fig. 2:**
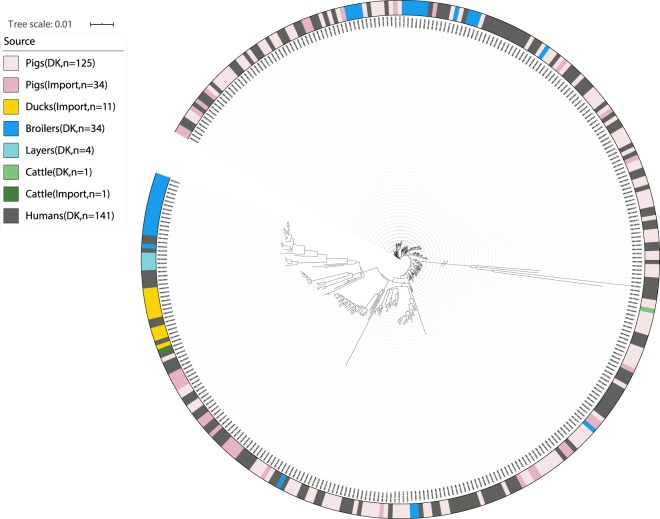
Phylogeny of the Danish dataset. //: Branch length to outgroup ST36 reduced by 30 from 0.71421 to 0.023807. Isolates are annotated by source. Light red: domestically produced pigs, Pigs(DK), light pink: imported pigs, Pigs(Import), yellow; imported ducks, Ducks(Import), blue: domestically produced broilers, Broilers(DK), turquoise: domestically produced eggs, Layers(DK), light green: domestically produced cattle, Cattle(DK), dark green: imported cattle, Cattle(Import), dark grey: Danish human salmonellosis cases, Humans(DK).

**Fig. 3:**
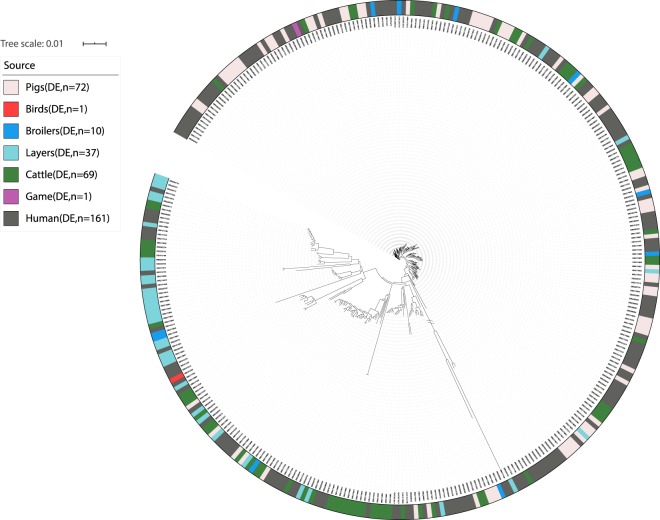
Phylogeny of the German dataset. //: Branch length to outgroup ST36 reduced by 30 from 0.73924 to 0.0246413333. Isolates are annotated by source. Light red: domestically produced pigs, Pigs(DE), red: domestically birds, Birds(DE), blue: domestically produced broilers, Broilers(DE), turquoise: domestically produced eggs, Layers(DE), dark green: domestically produced cattle, Cattle(DE), purple: domestically game, Game(DE), dark grey: German human salmonellosis cases, Human(DE).

**Fig. 4:**
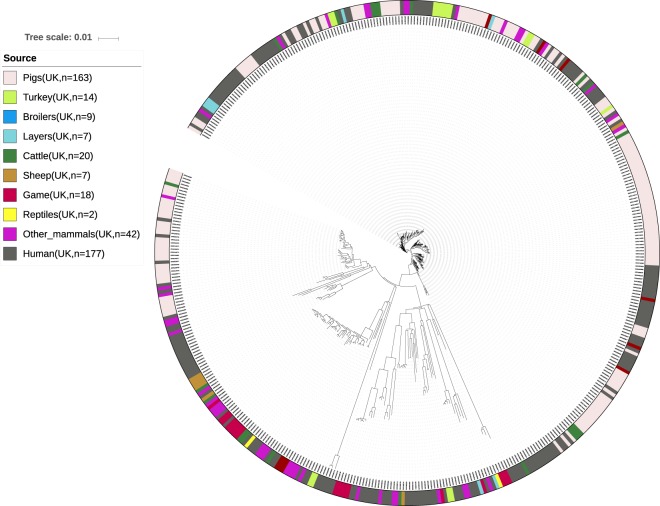
Phylogeny of the British dataset. Isolates are annotated by source. Light red: domestically produced pigs, Pigs(UK), light green: domestically produced turkey, Turkey(UK), blue: domestically produced broilers, Broilers(UK), turquoise: domestically produced eggs, Layers(UK), dark green: domestically produced cattle, Cattle(UK), brown: domestically produced sheep, Sheep(UK), red: Game(UK), yellow: reptiles(UK), purple: other mammals, dark grey: British human salmonellosis cases.

**Fig. 5:**
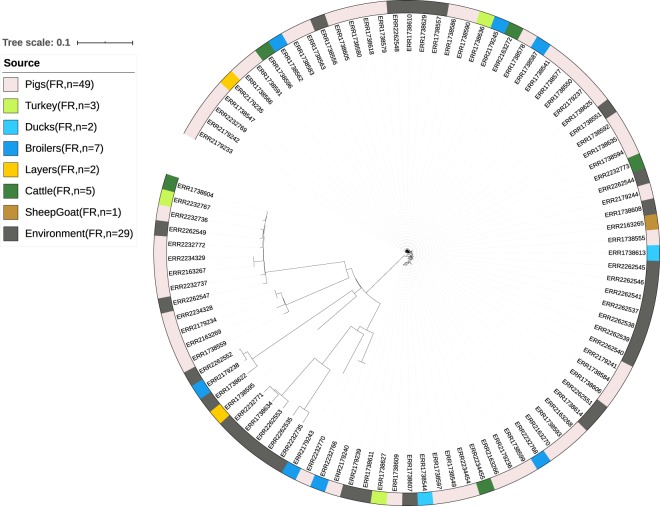
Phylogeny of the French dataset. Isolates are annotated by source. Light red: domestically produced pigs, Pigs(FR), green: domestically produced turkey, Turkey(FR), light blue: domestically produced ducks, Ducks(FR), blue: domestic produced broilers, Broilers(FR), orange: domestically produced eggs, Layers(FR), dark green: domestically produced cattle, Cattle(FR), brown: domestically produced sheep goats, SheepGoat(FR), purple: domestically produced crustaceans, Crustaceans(FR), dark grey: French environment samples, Environment(FR).

The applicability of the four datasets to develop new source attribution models was assessed by examining the population structure obtained from the phylogenetic analysis (Figs. 2, 3, 4 and 5).

From the population structure of the Danish, German and British datasets, human isolates were seen to be intermixed with the potential food and animal sources. From the population structure of the French dataset environmental isolates were seen to be intermixed with the potential sources of contamination. It was therefore concluded that all four datasets were applicable for development of new source attribution models.

## Usage Notes

All four datasets and a selection of the metadata provided here are available for download through ENA^26–29^ or SRA^30^. Accession numbers are listed in figshare^31^. The accession numbers are associated with larger projects and consequently include isolates not discussed in this descriptor. The curated datasets presented in this descriptor are described in figshare^31^.

These datasets were collected and sequenced with the purpose of developing new source attribution models based on sequencing data. This descriptor argues that all four datasets are applicable for this purpose. The four datasets can be used to further develop and benchmark source attribution models using new and emerging bioinformatics analysis and mathematical models. Next step in developing the new source attribution models could therefore be to apply further bioinformatics analyses to the datasets, such as core genome MLST, whole genome MLST and distance matrices and test these as input to statistical models such as machine learning and Bayesian based models as well as to population genetic methods such as the asymmetric island model, STRUCTURE and Network Analysis. This extensive work has already been initiated by the WP4/7 of COMPARE as conceptualized in Fig. 1.

## Data Availability

No code per se was developed for this article, as available tools were applied. The quality control pipeline used is available at bitbucket: https://bitbucket.org/RolfKaas/foodqcpipeline/ The CSI Phylogeny pipeline is available as a webtool through Center for Genomic Epidemiology (www.genomicepidemiology.org). Following options were used: Select min. depth at SNP positions: 10x. Select min. relative depth at SNP positions: 10%. Select minimum distance between SNPs (prune): 10. Select min. SNP quality: 30. Select min. read mapping quality: 25. Select min. Z-score: 1.96.
